# Potential risk of alpha-glucosidase inhibitor administration in prostate cancer external radiotherapy by exceptional rectal gas production: a case report

**DOI:** 10.1186/1752-1947-8-136

**Published:** 2014-05-05

**Authors:** Takuya Nishimura, Hideya Yamazaki, Kazuki Iwama, Yoshitaka Oota, Norihiro Aibe, Satoaki Nakamura, Ken Yoshida, Haruumi Okabe, Kei Yamada

**Affiliations:** 1 Department of Radiology, Ujitakeda Hospital, 24-1, Umonji, Uji-city, Kyoto 611-0021, Japan; 2 Department of Radiology, Graduate School of Medical Science, Kyoto Prefectural University of Medicine, 465 Kajii-cho, Kawaramachi Hirokoji, Kamigyo-ku, Kyoto 602-8566, Japan; 3 Department of Radiology, Osaka Medical College, 2-7 Daigakumachi, Takatsuki-City, Osaka 569-0801, Japan

**Keywords:** Tomotherapy, Alpha-glucosidase inhibitor, Prostate cancer, Internal organ motion

## Abstract

**Introduction:**

Radiotherapy is a standard treatment for prostate cancer, and image-guided radiotherapy is increasingly being used to aid precision of dose delivery to targeted tissues. However, precision during radiotherapy cannot be maintained when unexpected intrafraction organ motion occurs.

**Case presentation:**

We report our experience of internal organ motion caused by persistent gas production in a patient taking an alpha-glucosidase inhibitor. A 68-year-old Japanese man with prostate cancer visited our institution for treatment with helical tomotherapy. He suffered from diabetes mellitus and took an alpha-glucosidase inhibitor. Routine treatment planning computed tomography showed a large volume of rectal gas; an enema was given to void the rectum. Subsequent treatment planning computed tomography again showed a large volume of gas. After exercise (walking) to remove the intestinal gas, a third scan was performed as a test scan without tight fixation, which showed a sufficiently empty rectum for planning. However, after only a few minutes, treatment planning computed tomography again showed extreme accumulation of gas. Therefore, we postponed treatment planning computed tomography and consulted his doctor to suspend the alpha-glucosidase inhibitor, which was the expected cause of his persistent gas. Four days after the alpha-glucosidase inhibitor regimen was suspended, we took a fourth treatment planning computed tomography and made a treatment plan without gas accumulation. Thereafter, the absence of rectal gas accumulation was confirmed using daily megavolt computed tomography before treatment, and the patient received 37 fractions of intensity-modified radiotherapy at 74Gy without rectal gas complications. In this case study, the alpha-glucosidase inhibitor induced the accumulation of intestinal gas, which may have caused unexpected organ motion, untoward reactions, and insufficient doses to clinical targets.

**Conclusions:**

We suggest that patients who are taking an alpha-glucosidase inhibitor for diabetes should discontinue use of that particular medicine prior to beginning radiotherapy.

## Introduction

Prostate cancer has become one of the most pronounced cancers among Western men, and radiotherapy plays an important role in its treatment. To achieve optimal outcomes without elevated toxicity, organ motion is an important consideration during radiotherapy [1]. If a tumor moves out of the irradiated field, local control rates decrease and adverse events simultaneously increase. Therefore, numerous efforts have been made to limit organ motion [2,3]. We installed helical tomotherapy (HT; TomoTherapy Inc., Madison, WI, USA), which allows the delivery of image-guided, intensity-modulated radiotherapy (IMRT), thereby providing both accuracy and precision. This technique avoids risks to the organs while precisely irradiating the planning target volume, and the setup error is reduced using routine megavolt computed tomography (MVCT) prior to treatment.

Acarbose is an alpha-glucosidase inhibitor (AGI) that has been approved for the treatment of type 2 diabetes mellitus (DM) [4,5]. Acarbose inhibits carbohydrate digestion, allowing excessive volumes of undigested carbohydrate to reach the colon. Bacterial fermentation of this carbohydrate produces intestinal gas, which can cause flatulence, abdominal pain, and unintended organ motion. We report our experience with a patient who took an AGI and developed excessive intestinal gas, which resulted in prohibitive target movement. To the best of our knowledge, this is the first report of the potential radiotherapeutic risks of AGI-induced intestinal gas production.

## Case presentation

**Figure 1:**
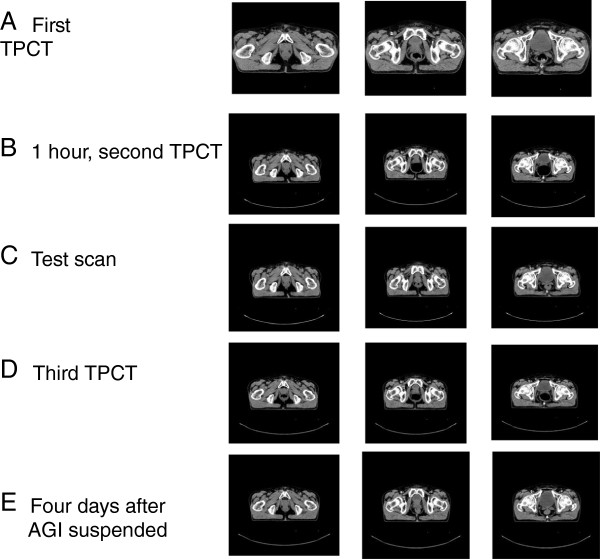
**Abdominal computed tomography images. (A)** Excessive gas production on initial treatment planning computed tomography images (the first scan). **(B)** After we gave him an enema, his rectum filled with gas (the second scan). **(C)** A test scan to confirm that his rectum is empty. **(D)** A few minutes later, the treatment planning computed tomography scan (the third) shows that more gas has accumulated. **(E)** Four days after cessation of the alpha-glucosidase inhibitor. TPCT, treatment planning computed tomography; AGI, alpha-glucosidase inhibitor.

A 68-year-old Japanese man presented to the urological outpatient department with high serum prostate-specific antigen levels (62.1ng/ml). Prostate biopsies revealed prostate cancer, with a Union for International Cancer Control (UICC) TNM classification and Gleason Score of cT2bN0M0 and 4+3, respectively. Our patient had had an acute myocardial infarction at 53 years of age; he had suffered from DM for 20 years, and he took insulin, metformin, famotidine, aspirin, ticlopidine hydrochloride, rosuvastatin calcium, and acarbose as medications. After neoadjuvant hormone therapy, he received IMRT using tomotherapy. One hour before treatment planning computed tomography (TPCT) using Aquilion 64 (Toshiba Medical Systems Corp., Tokyo, Japan), patients are instructed to empty their rectum but not their bladder. At the first TPCT scan, our patient’s rectum contained a large volume of gas (Figure 1A). Therefore, we conducted an enema and after saving urine again for approximately 1 hour, a second TPCT scan was performed (Figure 1B). Although enemas usually help void rectal gas, the second scan revealed a further increase in rectal gas. A short walk was advised, and rectum emptying was confirmed using a test CT scan (Figure 1C) prior to the third TPCT. Surprisingly, after only a few minutes, the third TPCT scan again showed rapid gas accumulation (Figure 1D). Thus, TPCT was postponed because of a high risk of exceptional intrafractional motion. Finally, we gave up taking TPCT that day. After consulting our patient’s doctor, his AGI regimen was suspended on the suspicion that it caused persistent gas accumulation. Four days later, his rectum function was normal (Figure 1E), only minimal intestinal gas was confirmed by daily MVCT, and 37 fractional IMRT were performed at 74Gy without gas incident.

## Discussion

Rectal gas is an important issue during radiotherapy for prostate cancer. A distended rectum significantly decreases local control because of systematic differences between planned and actual positions of the prostate during treatment and imprecision of tissue targeting [6,7]. Acarbose is an oral AGI that is used with diet and exercise programs to control high blood sugar in individuals with type 2 DM. Acarbose works by slowing the breakdown of starch (carbohydrates) from food into sugar, thereby inhibiting an increase in blood sugar levels after meals [4,5]. However, AGI leads to excessive gas production by bacterial fermentation of excessive undigested food in the gastrointestinal tract. The degree of gas production varies among patients. However, in this case, we observed dynamic rectal motions within minutes and attributed these to the influence of the AGI. During HT, adjustments for geometrical deviations of the prostate are achieved by moving the patient’s body and by confirming vacancy of the rectum before each session of radiotherapy. However, unintended abrupt rectal gas expansions result in insufficient doses to prostatic tumors and overexposure to normal rectal tissue, particularly the rectum, resulting in treatment failure and rectal bleeding.

Several authors have reported higher risks of late rectal toxicity in patients with DM [8-13]. DM causes damage to the microvasculature by inducing endothelial and vascular smooth muscle dysfunction [9]. The resulting delayed complications are related to ischemic injury and ulceration, owing to the loss of endothelial cell function and reduced proliferation of arterial and venous intima. Therefore, radiation-induced damage is prevalent in patients with DM. In addition, we suggest that AGI therapy results in enormous rectal gas accumulation, which elevates the risk of radiotherapy failure due to dynamic organ motion. Previously, this may have been overlooked because relatively few patients with DM take an AGI. Nonetheless, the use of AGIs should be monitored in patients with DM. In some institutes, CT scans are conducted only once for treatment planning and positioning because of limited time for linacgraphy. However, single CT scans neglect changes in rectal gas volumes that can cause migration of the prostate after planning. This case warrants further detailed studies of such risks using data from multiple institutions and a larger number of patients.

## Conclusions

AGIs can cause unintended exceptional intestinal gas production, which may induce critical prostate migration in prostate cancer external beam radiotherapy. Thus, patients with DM receiving pelvic radiotherapy should be asked whether they are taking an AGI prior to radiotherapy, and they should discontinue use of that particular medicine before beginning radiotherapy.

## Consent

Written informed consent was obtained from the patient for publication of this case report and any accompanying images. A copy of the written consent is available for review by the Editor-in-Chief of this journal.

## Abbreviations

AGI: alpha-glucosidase inhibitor; DM: diabetes mellitus; HT: tomotherapy; IMRT: intensity-modulated radiotherapy; MVCT: megavolt computed tomography; TPCT: treatment planning computed tomography.

## Competing interests

The authors declare that they have no competing interests.

## Authors’ contributions

TN and HY were major contributors in writing the manuscript. KI and YO analyzed and interpreted the patient data. NA, SN, KYo, KYa and HO followed up the patient. All authors read and approved the final manuscript.

## References

[B1] Langen KM Jones DTL Organ motion and its management Int J Radiat Oncol Biol Phys 2001 50 265 278 10.1016/S0360-3016(01)01453-5 11316572

[B2] Mutanga TF de Boer HC van der Wielen GJ Hoogeman MS Incrocci L Heijmen BJ Margin evaluation in the presence of deformation, rotation, and translation in prostate and entire seminal vesicle irradiation with daily marker-based setup corrections Int J Radiat Oncol Biol Phys 2011 81 1160 1167 10.1016/j.ijrobp.2010.09.013 21035957

[B3] Ogino I Uemura H Inoue T Kubota Y Nomura K Okamoto N Reduction of prostate motion by removal of gas in rectum during radiotherapy Int J Radiat Oncol Biol Phys 2008 72 456 466 10.1016/j.ijrobp.2008.01.004 18374517

[B4] Walton RJ Sherif IT Alberti KG Improved metabolic profiles in insulin-treated diabetic patients given an alpha-glucosidehydrolase inhibitor BMJ 1979 6158 220 221 10.1136/bmj.1.6158.220 PMC1597843 369651

[B5] Lardinois CK Greenfield MS Schwartz HC Vreman HJ Reaven GM Acarbose treatment of non-insulin-dependent diabetes mellitus Arch Intern Med 1984 144 345 347 10.1001/archinte.1984.00350140169023 6696573

[B6] Stasi M Munoz F Fiorino C Pasquino M Baiotto B Marini P Malinverni G Valdagni R Gabriele P Emptying the rectum before treatment delivery limits the variations of rectal dose-volume parameters during 3D-CRT of prostate cancer Radiother Oncol 2006 80 363 370 10.1016/j.radonc.2006.08.007 16959344

[B7] Nijkamp J Pos FJ Nuver TT de Jong R Remeijer P Sonke JJ Lebesque JV Adaptive radiotherapy for prostate cancer using kilovoltage con-beam computed tomography: first clinical results Int J Radiat Oncol Biol Phys 2008 70 75 82 10.1016/j.ijrobp.2007.05.046 17869445

[B8] Peeters ST Lebesque JV Heemsbergen WD Hart AA Koper PC Lebesque JV Localized volume effects for late rectal and anal toxicity after radiotherapy for prostate cancer Int J Radiat Oncol Biol Phys 2006 64 1151 1161 10.1016/j.ijrobp.2005.10.002 16414208

[B9] Akimoto T Muramatsu H Takahashi M Saito J Kitamoto Y Harashima K Miyazawa Y Yamada M Ito K Kurokawa K Yamanaka H Nakano T Mitsuhashi N Niibe H Rectal bleeding after hypofractionated radiotherapy for prostate cancer: correlation between clinical and dosimetric parameters and the incidence of grade 2 or worse rectal bleeding Int J Radiat Oncol Biol Phys 2004 60 1033 1039 10.1016/j.ijrobp.2004.07.695 15519772

[B10] Herold DM Hanlon AL Hanks GE Diabetes mellitus: a predictor for late radiation morbidity Int J Radiat Oncol Biol Phys 1999 43 475 479 10.1016/S0360-3016(98)00460-X 10078625

[B11] Skwarchuk MW Jackson A Zelefsky MJ Venkatraman ES Cowen DM Levegrün S Burman CM Fuks Z Leibel SA Ling CC Late rectal toxicity after conformal radiotherapy of prostate cancer (I): multivariate analysis and dose-response Int J Radiat Oncol Biol Phys 2000 47 103 113 10.1016/S0360-3016(99)00560-X 10758311

[B12] Vavassori V Fiorino C Rancati T Magli A Fellin G Baccolini M Bianchi C Cagna E Mauro FA Monti AF Munoz F Stasi M Franzone P Valdagni R Predictors for rectal and intestinal acute toxicities during prostate cancer high-dose 3DCRT: results of a prospective multicenter study Int J Radiat Oncol Biol Phys 2007 67 1401 1410 10.1016/j.ijrobp.2006.10.040 17241754

[B13] Cheung R Tucker SL Ye JS Dong L Liu H Huang E Mohan R Kuban D Characterization of rectal normal tissue complication probability after high-dose external beam radiotherapy for prostate cancer Int J Radiat Oncol Biol Phys 2004 58 1513 1519 10.1016/j.ijrobp.2003.09.015 15050331

